# Marking Embryonic Stem Cells with a 2A Self-Cleaving Peptide: A NKX2-5 Emerald GFP BAC Reporter

**DOI:** 10.1371/journal.pone.0002532

**Published:** 2008-07-02

**Authors:** Edward C. Hsiao, Yuko Yoshinaga, Trieu D. Nguyen, Stacy L. Musone, Judy E. Kim, Paul Swinton, Isidro Espineda, Carlota Manalac, Pieter J. deJong, Bruce R. Conklin

**Affiliations:** 1 Gladstone Institute of Cardiovascular Disease, San Francisco, California, United States of America; 2 Department of Medicine, University of California, San Francisco, California, United States of America; 3 Children's Hospital Oakland Research Institute, Oakland, California, United States of America; 4 Graduate Program in Pharmaceutical Sciences and Pharmacogenomics, University of California San Francisco, San Francisco, California, United States of America; 5 Department of Molecular and Cellular Pharmacology, University of California San Francisco, California, United States of America; City of Hope Medical Center, United States of America

## Abstract

**Background:**

Fluorescent reporters are useful for assaying gene expression in living cells and for identifying and isolating pure cell populations from heterogeneous cultures, including embryonic stem (ES) cells. Multiple fluorophores and genetic selection markers exist; however, a system for creating reporter constructs that preserve the regulatory sequences near a gene's native ATG start site has not been widely available.

**Methodology:**

Here, we describe a series of modular marker plasmids containing independent reporter, bacterial selection, and eukaryotic selection components, compatible with both Gateway recombination and lambda prophage bacterial artificial chromosome (BAC) recombineering techniques. A 2A self-cleaving peptide links the reporter to the native open reading frame. We use an emerald GFP marker cassette to create a human BAC reporter and ES cell reporter line for the early cardiac marker NKX2-5. NKX2-5 expression was detected in differentiating mouse ES cells and ES cell-derived mice.

**Conclusions:**

Our results describe a NKX2-5 ES cell reporter line for studying early events in cardiomyocyte formation. The results also demonstrate that our modular marker plasmids could be used for generating reporters from unmodified BACs, potentially as part of an ES cell reporter library.

## Introduction

Embryonic stem (ES) cells hold significant potential for studying the early developmental pathways of tissue differentiation and function. However, the identification and isolation of pure cell populations has been hampered by the heterogeneity of ES cell cultures and a paucity of robust genetic markers in ES cell lines. One popular approach for identifying gene expression in living tissues by microscopy or flow cytometry [1], [2] is with fluorescent proteins. In addition to the wide variety of colors, fluorescent markers with long [e.g., enhanced and emerald GFPs (EmGFP)] [3], [4] or short (e.g., destabilized GFP) [5], [6] half-lives are available.

Many current reporters achieve cell specificity by linking a small promoter fragment to a protein/reporter fusion, replacing an entire open reading frame with a reporter, or using an internal ribosomal entry sites (IRES) sequence to drive reporter expression. These constructs are not ideal for poorly characterized or large promoter regions. In addition, polycistronic constructs with IRES sequences can display differential expression of the individual cistrons [7], making it difficult to directly correlate promoter function to fluorescence levels.

Bacterial artificial chromosomes (BACs) of up to 300 kb have been used to create reporter constructs and transgenic animals [8], [9]. BAC reporters are capable of carrying large promoter and enhancer regions within a single construct. The long BAC arms also function to decrease integration-site effects in transgenic cell lines or mice. Rapid and efficient BAC modification techniques have been recently introduced [10], [11], and methods for creating GFP-based mouse BAC reporters for use in ES cells have also been described [12].

Ideally, reporter constructs should be easily created with different combinations of fluorophores and bacterial/eukaryotic selection markers linked to the same promoter region. This is particularly important when the reporters are to be used in different cell types or in cells from different species, since the efficiencies of the promoters driving selection markers vary [13]–[15]. In addition, peptide sequences added to fluorophores, such as GFP, may affect protein stability [6], [16] or organelle localization (e.g., if a signal sequence is attached), suggesting that a uniform fluorophore molecule may be beneficial when quantitative comparisons between different reporter lines are desired. Finally, modifications to the 5′ un-translated region and endogenous ATG start site may be undesirable since both of these regions are subject to chromatin modification [17] for transcriptional regulation.

The method presented here addresses these concerns and uses high-efficiency recombination techniques to minimize the need for unique reagents.

## Results

### Modular reporter cassettes

**Figure 1:**
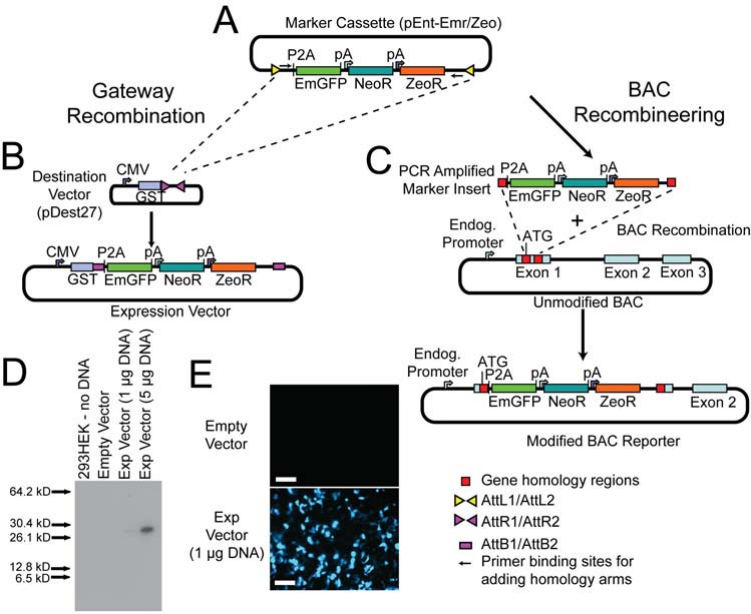
General strategy of the reporter cassette construct (pEnt-Emr/Zeo). (A) The fluorescent tag is composed of a 2A sequence preceding the EmGFP cistron. Separate bacterial and eukaryotic selection markers are also present. The entire three-cistron cassette is flanked by AttL sites for use in a Gateway recombination reaction (B) that introduces the construct into a destination vector (such as pDest27) with the corresponding AttR sites. In addition, primer binding sites are included for introducing the cassette into other vectors, such as BACs, by direct recombineering (C). (D) The 2A site is fully functional when an expression vector generated by LR Gateway insertion of pEnt-Emr/Tet into pDest27 (pExp/pD27/pEnt-Emr; Fig. S2) is introduced into 293HEK cells at low (1 µg) or high (5 µg) DNA doses. Complete separation of the GST tag and EmGFP polypeptides is detected by western blotting with a primary antibody against GFP. The expected size of the EmGFP polypeptide is 27 kD; unprocessed polypeptide retaining the GST tag would have appeared at 54 kD. (E) EmGFP fluorescence imaging of 293HEK cells transfected with empty pDest27 vector (top panel) or with 1 µg of expression vector (pExp/pD27/pEnt-Emr). White scale bar represents 100 µm.

To achieve our goal of a flexible, modular method for making fluorescent reporters, we designed a system that uses standardized marker cassettes that can be inserted in frame with a coding sequence for use in human or mouse cells (Fig. 1A–C). Each genetic component of the cassette (reporter, eukaryotic selection marker, and bacterial selection marker) is flanked by unique restriction sites to facilitate exchange with other components (Fig. S1). Variations of the cassette with different markers were generated (Table 1) to allow different combinations of fluorophores and selection markers. Although single-cistron dual selection systems are available (e.g., kanamycin/neomycin selection), we believe that our strategy of separate prokaryotic/eukaryotic selection cassettes allows for more flexibility and facilitates the creation of multi-color reporter cells.

**Table 1:** Series of marker cassettes for BAC reporters.

Name	Fluorophore	Bacterial Selection	Eukaryotic Selection	Notes
pEnt-Emr/Tet	Emerald GFP	Tetracycline	SV40-Neo	Also KanR
pEnt-Emr/Zeo	Emerald GFP	Zeocin	SV40-Neo	Also KanR
pEnt-Emr/Amp	Emerald GFP	Ampicillin	SV40-Neo	Also KanR
pEnt-mCherry	mCherry	Tetracycline	SV40-Neo	Also KanR
pEnt-mOrange	mOrange	Tetracycline	SV40-Neo	Also KanR
pEnt2-mCherry	mCherry	Zeocin	Rex-Neo	Rex-Neo is flanked by FRT sites. Carries ccdB gene.

Maps for these marker cassette plasmids appear in Fig. S1.

A 2A ribosomal skip site links the N-terminal peptide fragment of the endogenous gene to the introduced marker. The self-cleaving 2A site generates two separate peptides in equal concentrations [18], [19] via a ribosomal “skip” mechanism just before the C-terminal end of the 2A peptide [20]. This feature has several advantages over traditional fusion proteins. The fluorophore that is released would carry the same N-terminal modification between different reporters constructs. For example, all reporters generated using the pEnt-Emr cassette (Figure S1A) will have a single proline added to the N-terminus of EmGFP. This uniform modification will help minimize the potential for any localization, spectral, or stability differences that could arise with different N-terminal modifications in a traditional GFP fusion strategy [6], [16]. The 2A strategy also ensures that a functional fluorophore molecule is released, since not all N-terminal GFP fusion proteins work [3]. Finally, our strategy preserves the regulatory sequences surrounding the promoter, 5′ un-translated region, and endogenous ATG start site, since these regions may be subject to chromatin modification [17] for transcriptional regulation. This approach is especially beneficial for studies of genes in which the ATG start site has been identified, but the structure of the open reading frame has not been defined.

To facilitate integration of the marker cassette into a target site, the cassette is flanked by primer binding sites for BAC recombineering with the lambda prophage *Escherichia coli* system [10], as well as AttL1 and AttL2 sequences for recombination with the Gateway system [21], if corresponding target AttR sites have been introduced in advance. Both of these modalities were included because Gateway recombination is an elegant method for smaller target constructs (<50 kb in our hands) while the lambda prophage system allows modification of larger targets [10], [11].

Our prototype marker construct, pEnt-Emr (Fig. S1), utilizes EmGFP. This long half-life fluorophore is ideal for detecting faint signals but has lower temporal sensitivity than shorter half-life versions of GFP [3]. The EmGFP is preceded by the 2A self-cleaving peptide sequence. The construct contains the eukaryotic SV40-neomycin selection marker and a bacterial selection marker, either for tetracycline (pEnt-Emr/Tet, Fig. S1A) or for Zeocin (pEnt-Emr/Zeo, Fig. S1B).

To demonstrate that the 2A site is functional in our construct, the pEnt-Emr/Tet cassette was recombined by the Gateway method into the pDest27 expression vector to generate the expression vector pExp/pD27/pEnt-Emr/Tet (Fig. S2). Protein obtained from HEK293 cells transfected with pExp/pD27/pEnt-Emr showed no detectable uncleaved product by western blot with an antibody against GFP (Fig. 1D). Fluorescence microscopy showed strong expression of EmGFP within the cells (Fig. 1E).

### Creating a NKX2-5 Emerald GFP BAC reporter

We next demonstrated the utility of the pEnt-Emr marker cassette for BAC recombineering. We used the pEnt-Emr/Zeo plasmid to create a BAC reporter ES cell line for the NKX2-5 gene, a marker of early cardiomyocyte differentiation. The NKX2-5 locus is particularly challenging: although the 5′ enhancer fragments were characterized earlier [22] and mouse NKX2-5 BAC reporters have been used to create transgenic mice [23], the NKX2-5 base promoter was only recently described [24]. Early NKX2-5 reporters used a hybrid construct of an NKX2-5 enhancer with a generic promoter, such as HSP68 [25]. While these hybrid reporters could recapitulate the tissue-specific expression of NKX2-5, the hybrid enhancer-promoter made it difficult to use the reporter as a measure of endogenous expression levels.

**Figure 2:**
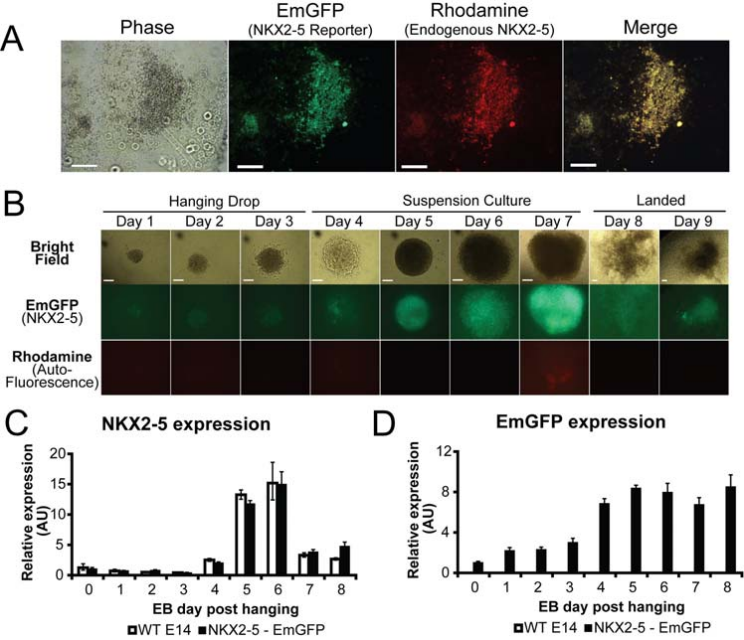
Characterization of murine ES cells carrying the RP11-88L12 NKX-2.5-EmGFP BAC reporter. (A) Mouse E14-NKX2-5 ES cells were differentiated into embryoid bodies containing cardiomyocytes by the hanging-drop method. Representative close-up photos of a beating region from NKX2-5 BAC clone 2 are shown. Day 11 embryoid bodies were examined for co-localization of the NKX2-5 emerald GFP reporter (EmGFP panel) with endogenous NKX2-5 (rhodamine panel). NKX2-5 linked emerald GFP expression was strongly correlated with regions containing beating cells (Movies S1 and S2) with minimal auto-fluorescence (Movie S3), consistent with a cardiomyocyte fate. White scale bar indicates 100 µm. (B) Fluorescence microscopy timecourse of embryoid body formation shows low levels of emerald GFP at day 4 of differentiation and an easily visible signal by day 7 (middle row). No significant auto-fluorescence of the embryoid bodies was detected when viewed through the rhodamine filter set (bottom row). White scale bars on bright field images indicate 100 µm (top row). Representative photographs from a total of 14 embryoid bodies analyzed at each timepoint are shown. (C) Quantitative PCR analysis of the E14-NKX2-5-EmGFP cells and wild type E14 cells for NKX2-5 RNA levels during cardiomyocyte formation showed that the reporter does not affect NKX2-5 RNA levels. NKX2-5 mRNA levels started to increase at day 4 and were highest at days 5–6. (D) Quantitative PCR analysis of pooled E14-NKX2-5-EmGFP EBs or wild type E14 EBs shows that EmGFP is detectable only in the E14-NKX2-5-EmGFP cell line and that EmGFP levels rise at day 4; however, the EmGFP mRNA levels persist at days 7 and 8, even when NKX2-5 RNA levels decrease, consistent with the known long half-life of EmGFP mRNA. Error bars represent+/−1 SD of technical triplicates from pooled EB samples. The differentiation timecourse and qPCR analysis were performed in duplicate.

We selected the human BAC RP11-88L12 containing the NKX2-5 locus and large flanking regions to make a reporter that would function in both mouse and human cells (Fig. S3A). A 3.6-kb PCR fragment amplified from pEnt-Emr/Zeo containing the marker cassette and 50 bp of flanking sequence was recombined into the open reading frame of the NKX2-5 locus 26 amino acids downstream of the native ATG site (Fig. S3B, C). The modified BAC (RP11-88L12 NKX2-5-EmGFP) was verified by restriction digest and by sequencing of the recombineering junctions before electroporation into the E14 mouse ES cell line and selection with neomycin. Three positive clones were identified and characterized by immunohistochemistry for NKX2-5 after *in-vitro* differentiation into beating cardiomyocytes. Two clones showed similar immunohistochemical co-localization of EmGFP with NKX2-5 in day 11 embryoid bodies (EBs); the third clone showed no EmGFP expression. One of the first two clones was arbitrarily chosen for further characterization (Fig. 2A).

### Characterization of NKX2-5 expression in mouse ES cells

**Figure 3:**
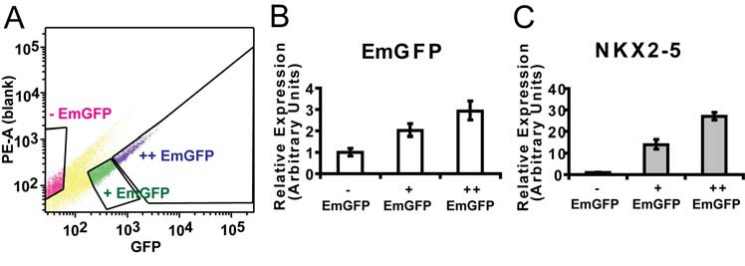
Quantitative PCR analysis of NKX2-5 marked E14 cells after fluorescence activated cell sorting (FACS). (A) Embryoid bodies formed by suspension culture from the NKX2-5-marked E14 ES cell line were FACS sorted into negative (−EmGFP; 13.9%), intermediate (+EmGFP; 14.5%), and high EmGFP (++EmGFP; 5.8%) expressing fractions at day 9. (B) Expression levels of EmGFP mRNA in FACS-sorted cell populations. (C) Expression levels of NKX2-5 in FACS-sorted cell populations, showing that NKX2-5 mRNA levels are increased in cells with higher EmGFP mRNA levels. The FACS analysis was performed in triplicate. Error bars represent+/−1 SD of technical triplicates.

We used the NKX2-5 marked ES cell line to examine NKX2-5 expression during ES cell differentiation into cardiomyocytes. By fluorescence microscopy, NKX2-5 expression was first detected at day 4 of EB formation and peaked around day 7 (Fig. 2B). EmGFP expression correlated with areas of beating cells (Movie S1, S2 and S3). We observed some EmGFP fluorescence in a small number of cells that were not beating, consistent with known NKX2-5 expression in non-cardiac cell types [22], [23]. Quantitative PCR analysis on whole EBs confirmed that initial expression of the EmGFP reporter coincided with that of the endogenous NKX2-5 (Fig. 2C). However, mRNA levels and fluorescence of EmGFP remained elevated after NKX2-5 expression had decreased (Fig. 2D), consistent with the known long mRNA and protein half-life of EmGFP [3]. Fluorescence-activated cell sorting (FACS) with the EmGFP marker on dissociated EBs showed that we could purify NKX2-5-positive cells based on EmGFP fluorescence (Fig. 3). These cells have been used to show that canonical Wnt signaling can positively regulate cardiogenesis [26].

**Figure 4:**
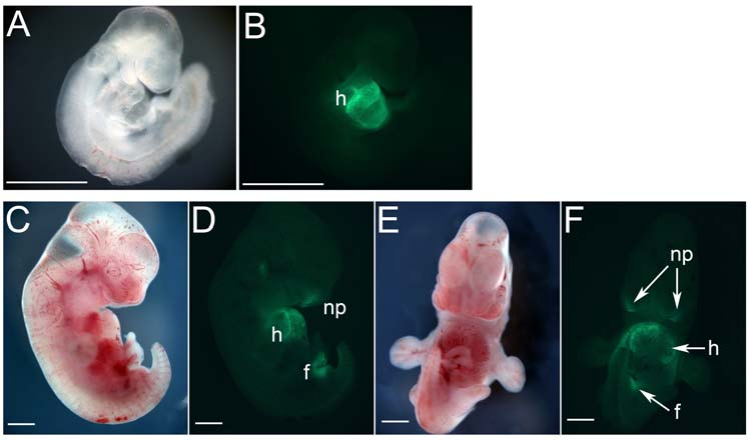
Transgenic embryos generated by the laser-ablation method show expression of NKX2-5 in the heart and foregut. (A, B) Right-sided view of a mouse e9.5–10 embryo showing strong expression of NKX2-5-EmGFP in the folding heart tube (h). (C, D) Right-sided and (E, F) anterior views of a mouse e12.5–13 embryo showing expression of NKX2-5 EmGFP in the heart (h) and foregut/stomach (f), as well as faint signal in the nasal pits (np). 1-mm scale bars are indicated in white.

To confirm that the NKX2-5 EmGFP reporter localized expression of NKX2-5 in an endogenous setting, we created chimeric mice from the NKX2.5 marked ES cells with an eight-cell laser-assisted technique to maximize embryo chimerism [27], [28]. Chimeric embryos were harvested at e9.5–10 and e13.5–14 days and examined for EmGFP fluorescence. Representative chimeric embryos (Fig. 4) show that EmGFP is strongly localized to the developing heart tube in e9.5–10 embryos and is present in the heart and foregut by e13.5–14, consistent with reported expression patterns of NKX2-5 [22]–[24]. In addition, we observed very weak EmGFP signals within the nasal placodes. Similar results were obtained in chimeric mice made by traditional blastocyst injection techniques, although with lower degrees of chimerism.

## Discussion

We created a modular reporter system that uses recombineering techniques to introduce a fluorophore/selection cassette into the open reading frame of a protein via a 2A self-cleaving peptide sequence. Our results demonstrate the system's functionality by creating an ES cell line and mouse chimeras carrying a NKX2-5 EmGFP BAC reporter for marking early cardiomyocytes. The results also show that EmGFP parallels NKX2-5 expression in both ES cells and transgenic chimeric mice, although temporal assessment of NKX2-5 expression was limited by the long fluorescence half-life of EmGFP.

The modular structure of our reporter cassette allows different fluorophores to be used for future constructs, including mCherry or mOrange [1] (Table 1) or destabilized forms of GFP. In addition, we created a marker cassette for making future reporters for human stem cells. Although the SV40-neomycin selection cassette has been used in human and mouse cells, viral promoters display varying abilities to express reporter constructs in different cell types [13], [29]. The pEnt2-mCherry construct contains a neomycin selection marker driven by the Rex-1 promoter, which is active only in undifferentiated ES cells [30], [31] (Fig. S1F). Both the eukaryotic and prokaryotic selection markers are also flanked by FRT sites for optional excision by FLP recombinase. This series of constructs will be particularly useful for creating reporters from unmodified human BACs and complement existing resources for mouse BAC reporters [12].

The modular system described here will allow rapid generation of libraries of genetic markers for expanded applications in flow cytometry, gene expression studies, and ES cell differentiation studies. The modular design allows a wide variety of reporter molecules to be used, including those with long or short half-lives to maximize detection sensitivity or temporal fidelity, respectively. In addition, these modular constructs will allow rapid introduction of different combinations of reporters and selection markers for use in different cell types and for testing constructs in mouse ES cells before introduction into human ES cell lines. The 2A site can be used to create reporter constructs that preserve the regulatory sequences at the 5′ end of the open reading frame. Finally, the use of a 2A ribosomal skip sequence is a viable method of linking multiple polypeptides together in a single cistron, particularly for use in ES cells [32]. This method will be useful for co-expression of different reporter and effector molecules, such as the tetracycline transactivator, Cre recombinase, additional antibiotic selection markers, or an engineered receptor [33]–[35]. We are hopeful that this system will facilitate rapid development of multifunctional expression constructs for creating reporter ES cell lines.

## Materials and Methods

### pEnt-Emr reporter cassette plasmids

A generalized strategy for the reporter construct method is presented in Fig. 1. Briefly, the pEnt-Emr/Tet reporter cassette (Fig. S1A) was created in the Invitrogen (Carlsbad, CA) Gateway entry vector plasmid pEntr2B. PCR cloning was used to introduce the 2A sequence (P2A version), EmGFP, and pA sequences in frame with the AttL1 site. The SV40-neomycin-pA cassette, PCR amplified from pDest27 (Invitrogen), was included for eukaryotic selection; a tetracycline-resistance cassette from pBR322 [36] was included for bacterial selection. The individual cassettes were separated by unique restriction sites, allowing easy swapping of individual components (Fig. S1A). Because of technical concerns that the tetracycline resistance gene could interfere with selection of the modified BACs, we created the pEnt-Emr/Zeo reporter cassette (Fig. S1B) by inserting a zeocin-resistance cassette from pDoner/Zeo (Invitrogen) in place of the tetracycline-resistance cassette. Derivative vectors with mCherry or mOrange [1] or the Rex-1 promoter [30], [31] are shown in Table 1 and Fig. S1C–F. All sequence maps were generated using VectorNTI 10 (Invitrogen). The reporter constructs in Table 1 and the RP11-88L12 NKX2-5-EmGFP (described below) can be obtained from the BACPAC Resources Center at the Children's Hospital Oakland Research Institute (http://bacpac.chori.org) or by emailing bacpacorders@chori.org.

### pExp/pD27/pEnt-Emr expression vector

The pExp/pD27/pEnt-Emr expression vector (Fig. S2) was created by Gateway recombination of the pEnt-Emr/Tet reporter cassette (entry vector) with the pDest27 (Invitrogen) destination vector using LR Clonase II (Invitrogen), according to the manufacturer's instructions.

### Western blot and microscopy

For the western blot experiments, 1 or 5 µg of pExp/pD27/pEnt-Emr or 1 µg of empty pDest27 was introduced into 293HEK cells with lipofectamine (Invitrogen), according to the manufacturer's instructions. EmGFP fluorescence was confirmed 24 h later by microscopy, and whole-cell protein extract was obtained by lysis in RIPA buffer (50 mM Tris-HCl, pH 7.4, 150 mM NaCl, 1% Triton X-100, 0.1% SDS) with protease inhibitor cocktail (Complete, Mini, EDTA-free, Roche Molecular Biochemicals, Indianapolis, IN). After quantitation of protein content by the D_c_ protein assay kit (BioRad Laboratories, Hercules, CA), 60 µg of protein for each sample was separated on a NuPAGE 12% Bis/Tris MOPS buffer protein gel (Invitrogen) and blotted onto nitrocellulose membrane (0.45-µm pore) as directed by the manufacturers. The western blot was probed using a 1∶750 dilution of anti-GFP antibody (BD Biosciences #632375, San Jose, CA), a 1∶10,000 dilution of anti-mouse-HRP secondary antibody (Amersham/GE Healthcare NA931, Piscataway, NJ), and detected using the SuperSignal West Pico Chemiluminescent Kit (Pierce, Rockford IL). All images were captured on a Zeiss Axiovert 200M inverted microscope equipped with an Axiocam HRC camera and GFP reflector cubes.

### BAC modification

The RP11-88L12 (chr5: 172,467,260–172,659,285 in NCBI Build 36.1) human BAC (RP11 library, BACPAC Resources) containing the NKX2-5 open reading frame with 60 kb of upstream and 130 kb of downstream sequences was selected for modification with a variant of the BAC recombineering method [10]. The BAC ends were reconfirmed by BAC end sequencing. The marker component of pEnt-Emr/Zeo was amplified using long-range PCR with the forward and reverse primers containing 50 nt of homology sequence (Table 2A). The PCR product and BAC were introduced into the heat-sensitive recombinase strain DY380. Recombined BAC candidates selected by Zeocin resistance were screened by PCR for correct 5′ and 3′ recombination events (Table 2B and C) and confirmed by NotI digest on a pulse-field gel electrophoresis (CHEF-DRIII, BioRad) to check for deletion mutants. The final BAC was named RP11-88L12 NKX2-5-EmGFP (Fig. 1; Fig. S3). The final construct was sequenced from the marker cassette outwards towards the genomic sequence, using the forward and reverse cassette primers (Table 2B) to confirm the correct reading frame and integration site of the marker cassette.

**Table 2:** Primer Sequences.

**A. BAC Recombineering primers for NKX2-5 into pEnt-Emr/Zeo**		
NKX2-5 Forward	*CCCTTCTCAGTCAAAGACATCCTAAACCTGGAACAGCAGCAGCGCAGCCT* **GAACCAATTCAGTCGACAAT**	3.36 kb
NKX2-5 Reverse	*TGTTTCCTCCTCACCTTTCTTTTCGGCTCTAGGGTCCTTGGCTGGGTCGG* **TCAGTGGTGACACTGGTTC**	
**B. BAC Screening primers (5′ Junction)**		
NKX2-5 Genomic Forward	ACCTGGCGCTGTGAGACT	362 bp
Marker Cassette Reverse	CAGATGAACTTCAGGGTCAG	
**C. BAC Screening primers (3′ Junction)**		
Marker Cassette Forward	AGGACTGAGAATTCGAACG	367 bp
NKX2-5 Genomic Reverse	GTTTCTTGGGGACGAAAG	
**D. Sybr Green primers**		
NKX2-5-F2	CAAGTGCTCTCCTGCTTTCC	136 bp
NKX2-5-R2	GGCTTTGTCCAGCTCCACT	
EmGFP-F2	AGCAAAGACCCCAACGAGAA	60 bp
EmGFP-R2	GGCGGCGGTCACGAA	
ActB-F1	TTGCTGACAGGATGCAGAAG	141 bp
ActB-R1	ACATCTGCTGGAAGGTGGAC	

(A) BAC recombineering primers for PCR amplification of the marker cassette. Regions of homology to the NKX2-5 open reading frame are indicated in italics. Regions that are complementary to sequences within the pEnt-Emr/Zeo marker cassette are indicated in bold. (B, C) BAC screening primers for the 5′ and 3′ recombination junctions are listed. The marker cassette reverse sequencing primer binds within the EmGFP gene, and the marker cassette forward binds within the zeosin resistance gene. Both marker cassette primers can be used to for confirmation by sequencing. All primer sequences are listed 5′ to 3′. Product sizes are listed in the right-most column. (D) SybrGreen primers used for Figure 2 of this study.

### NKX2-5 BAC reporter ES cells

Feeder-independent mouse ES cells (129/OlaHsd strain, subline E14Tg2A.4) were maintained in normal growth media supplemented with murine leukemia inhibiting factor as described [37]. RP11-88L12 NKX2-5-EmGFP BAC DNA (10 µg) was linearized with the homing enzyme PI-SceI (New England Biolabs, Ipswich, MA), which cuts once in the pBAC-e3.6 backbone of the RP11-88l12 BAC. The DNA was electroporated into 3×10^6^ ES cells using a BioRad Gene Pulser XCell at 800 V, 10 µF, and T**_c_** = 0.3. ES cell cultures were selected in normal growth media [37] supplemented with 175 ng/ml neomycin (Gibco BRL/Invitrogen, Carlsbad, CA) for 10 days. Four robustly growing colonies were identified, and three were subsequently identified as carrying the BAC transgene by PCR screening for the 5′ junction (Table 2B). Two of these cell lines showed similar immunohistochemistry results, and line 2 was arbitrarily chosen for the remaining experiments. The third line did not show EmGFP expression.

ES cells were differentiated into cardiomyocytes by the hanging drop method as described [38], [39]. Briefly, 20-µl droplets of differentiation medium (ES cell growth medium [37] without leukemia inhibitory factor and supplemented with 20% FBS), containing 500 mouse ES cells were suspended upside-down for 2 days in V-bottom 96-well plates, causing the cells to aggregate into EBs. EBs were maintained in suspension cultures for 5 days and then plated onto gelatin-coated, 24-well or 96-well tissue-culture plates (one EB per well), or onto gelatin-coated glass cover-slips. The medium was replaced every 2–3 days. By day 8 of differentiation, clusters of myocytes within the EBs could be observed contracting spontaneously. Immunohistochemistry and video microscopy was done on EBs after 11 days of differentiation.

For analysis of RNA expression, total RNA from the EBs was isolated using RNAStat-60 (Iso-Tex Diagnostics, Friendswood, TX), according to manufacturer's instructions. Quantitative PCR analysis of mRNA levels was done on an Applied Biosystems (Foster City, CA) 7900HT real-time thermocycler with SybrGreen primers for NKX2-5 and EmGFP [40] and normalized to beta actin levels. The NKX2-5 and beta actin primers were designed using Primer3 [41] as described [42].

For the FACS experiments, EBs were formed from NKX2-5-marked E14 ES cells with a suspension culture system as described [26]. The suspension culture helps minimize cell losses that may occur when EBs are dissociated from adhesion cultures. Briefly, ES cells were propagated in maintenance medium (Glasgow MEM, Sigma-Aldrich, St. Louis, MO) supplemented with 10% FBS (HyClone, Logan, UT), 1 mM 2-mercaptoethanol (Sigma), 2 mM L-glutamine (Gibco-BRL), 1 mM sodium pyruvate (Gibco-BRL), 0.1 mM minimum essential medium containing nonessential amino acids (Gibco-BRL), and leukemia inhibitory factor (LIF)-conditioned medium (1∶1,000). EBs were formed by culturing ESCs (6×10^5^ per well) for 3 days in ultra-low attachment six-well plates (Corning, Lowell, MA) in differentiation medium (DM) with the same components as maintenance medium but with 20% FBS and no LIF. EBs were maintained in suspension culture until dissociation with trypsin at day 9. Individual cells were sorted on a fluorescence-activated cell sorter (FACS DiVa, BD Biosciences) into negative, intermediate, and high EmGFP fluorescence fractions (Figure 3A). Total mRNA was isolated using RNAStat-60, and quantitative PCR analysis was done using mouse Taqman primers for NKX2.5 (ABI Mm00657783_m1), beta actin (ABI Mm00607939_s1) and GFP [43].

### Immunohistochemistry

EBs landed on gelatin-coated cover slips at day 7 of the cardiomyocyte differentiation protocol were allowed to grow until day 11, when clear beating regions could be identified. Immunohistochemistry was performed using the rabbit NKX2-5 H114 primary antibody (sc-14033, 1∶250 dilution, Santa Cruz Biotechnology, Santa Cruz, CA) and an Alexa-594 goat-anti-rabbit secondary antibody (A11037, 1∶250 dilution, Molecular Probes/Invitrogen). Immunohistochemistry controls for auto-fluorescence and background staining are shown in Fig. S4. All images were captured on a Zeiss Axiovert 200M inverted microscope equipped with an Axiocam HRC camera and GFP and rhodamine reflector cubes.

### Chimeric mice

Mouse chimeras were generated by the Gladstone Transgenic/Gene Targeting Core facility by injection of E14 ES cells carrying the RP11-88L12/NKX2-5-EmGFP transgene into eight-cell embryos assisted by a Xyclone laser system (Hamilton Thorne Biosciences, Beverly, MA), as described [28], to maximize chimerism for analysis of F0 progeny [27]. Forty-eight embryos were implanted in four surrogate mothers. Two of three embryos recovered at e12.5-13, and three of 14 embryos recovered at e9.5-10, were positive for cardiac EmGFP expression by whole-embryo fluorescence microscopy on a Leica MZLLIII dissecting microscope. Images were captured using a Zeiss Axiocam camera with GFP filters.

## Supporting Information

Figure S1 Maps of the different marker constructs with major features indicated. (A) pEnt-Emr/Tet and (B) pEnt-Emr/Zeo used in this manuscript. Unique restriction sites are noted in maroon, and restriction sites with more than one recognition sequence are in black. Additional marker cassettes have also been created, including (C) pEnt-Emr/Amp, (D) pEnt-mCherry, (E) pEnt-mOrange, and (F) pEnt2-mCherry. Note that pEnt2-mCherry contains a ccdB gene to increase the yield of correct targets after recombineering. In addition, a human Rex-1 promoter is used to drive the neomycin resistance gene. Both the eukaryotic and prokaryotic selection markers are flanked by FRT sites to allow optional excision.
(0.07 MB PDF)

Figure S2 Map of pExp/pD27/pEnt-Emr expression vector indicating major features. Unique restriction sites are indicated in maroon, and restriction sites with more than one recognition sequence are in black.
(0.02 MB PDF)

Figure S3 Maps of the modified region from the RP11-88L12 NKX2-5 BAC (RP11-88L12 NKX2-5-EmGFP). (A) Location of the human NKX2-5 open reading frame (blue, chr5: 172,591,744–172,594,868) within the RP11-88L12 BAC (black, chr5: 172,467,260–172,659,285 in NCBI Build 36.1). A second gene, BNIP1, was identified on RP11-88L12 by BAC-end sequencing (red, chr5: 172,504,146–172,523,950). (B) Enlargement of the modified NKX2-5 open reading frame showing insertion of the pEnt-Emr/Zeo marker cassette and locations of primers. (C) Sequence from the 5' junction showing locations of the 5' region of the NKX2-5 protein, 2A, primers, and emerald GFP.
(0.03 MB PDF)

Figure S4 Immunohistochemistry background controls. Representative photographs of wild type E14 (A–C) and E14-NKX2-5-EmGFP (D–F) embryoid bodies examined at day 7 of differentiation show that NKX2-5 EmGFP fluorescence is easily detectable in the E14-NKX2-5-EmGFP EB, but no EmGFP fluorescence is present in the wild type EB. In addition, minimal auto-fluorescence in the rhodamine channel is detected in both the wild type and E14-NKX2-5-EmGFP lines. Twelve EBs derived from each line were examined. (G–I) Immunohistochemistry with the Alexa-594 secondary antibody, but no NKX2-5 primary antibody, on day 8 E14-NKX2-5-EmGFP embryoid bodies (after landing and attachment onto coverslips) show diffuse but detectable EmGFP (as previously seen in Fig. 2B) and no background staining by the Alexa-588 secondary antibody. White scale bars indicate 100 µm.
(5.50 MB TIF)

Movie S1 GFP-fluorescence Quicktime movies of an NKX2-5-EmGFP fluorescent beating area. Beating ES cell derived cardiomyocytes (d11 after hanging) show EmGFP fluorescence. Phase microscopy images of the same beating region is shown in Movie S2. Rhodamine filter images to determine regions of auto-fluorescence are shown in Movie S3.
(8.13 MB MOV)

Movie S2 Phase image Quicktime movies of an NKX2-5-EmGFP fluorescent beating area. Beating ES cell derived cardiomyocytes (d11 after hanging) showing EmGFP fluorescence (Movie S1) visualized here with phase microscopy.
(11.70 MB MOV)

Movie S3 Rhodamine-fluorescence Quicktime movies of an NKX2-5-EmGFP fluorescent beating area. Beating ES cell derived cardiomyocytes (d11 after hanging) showing EmGFP fluorescence (Movie S1) are visualized here with phase microscopy to demonstrate minimal auto-fluorescence.
(3.15 MB MOV)
