# Supplementary figures and images for: Marking Embryonic Stem Cells with a 2A Self-Cleaving Peptide: A NKX2-5 Emerald GFP BAC Reporter
Source: PLoS One. 2008 Jul 2;3(7):e2532. doi: 10.1371/journal.pone.0002532 (PMC2430532; doi:10.1371/journal.pone.0002532)

Figure S1A

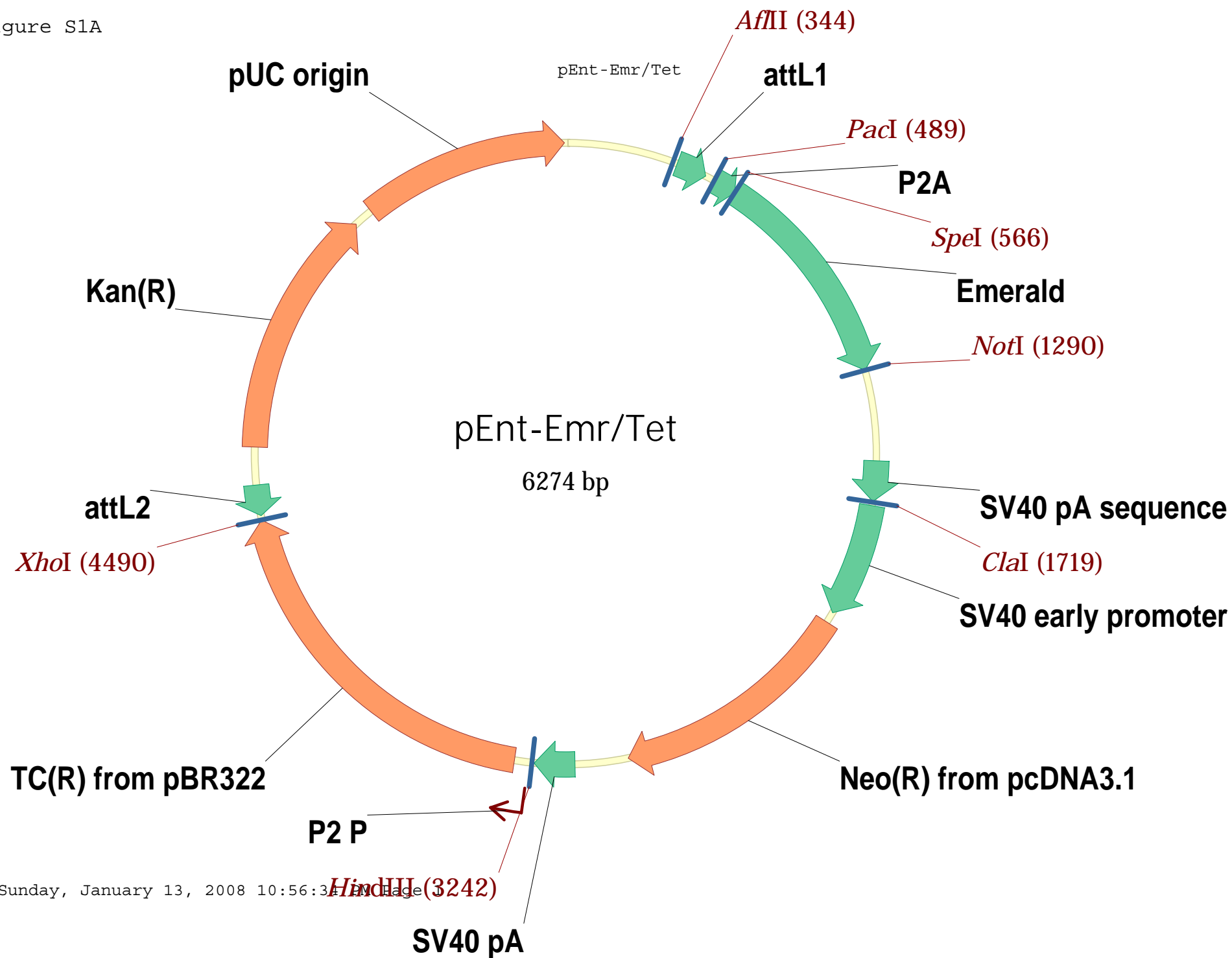

Figure S1B

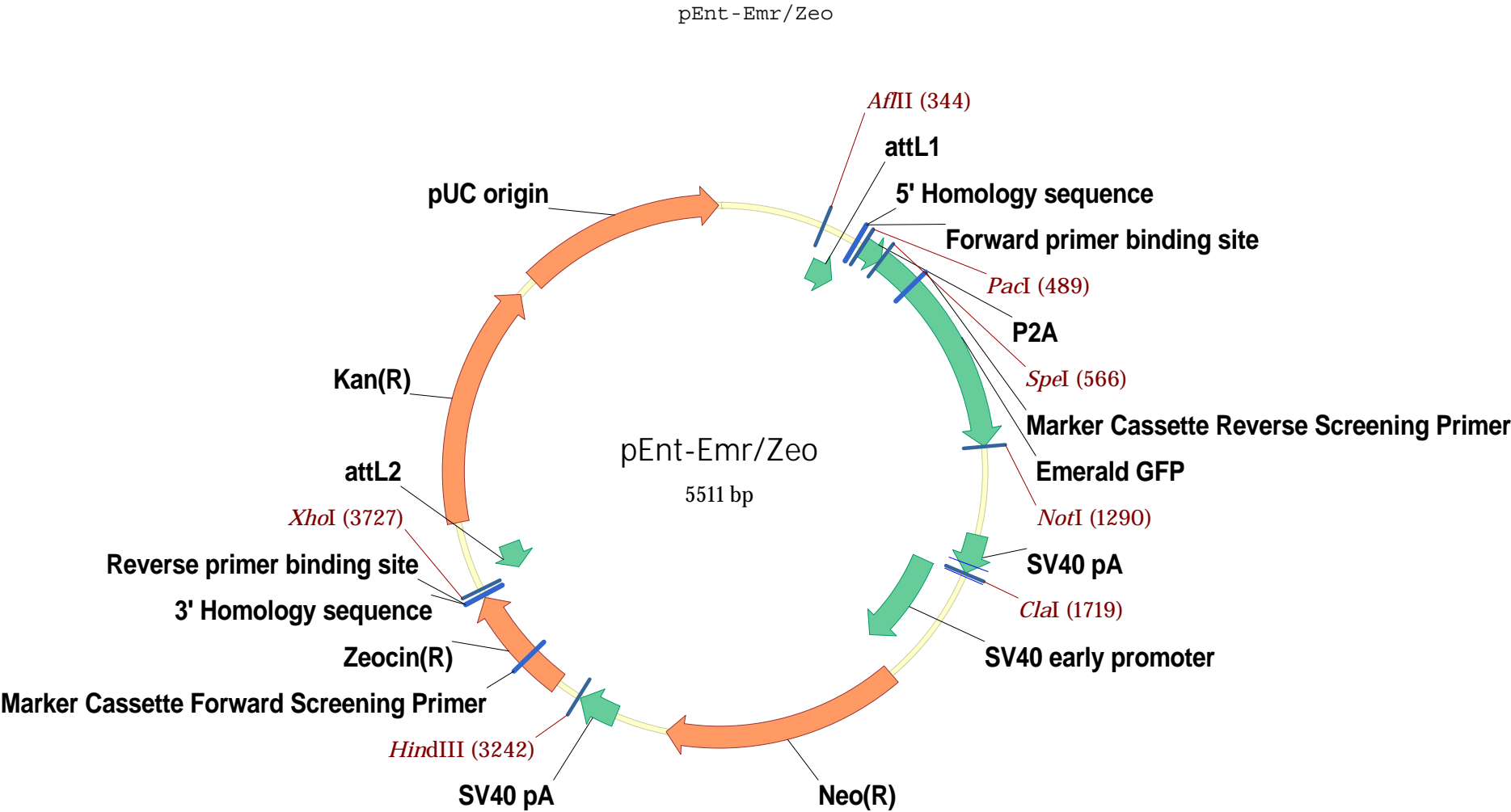

Figure S1C

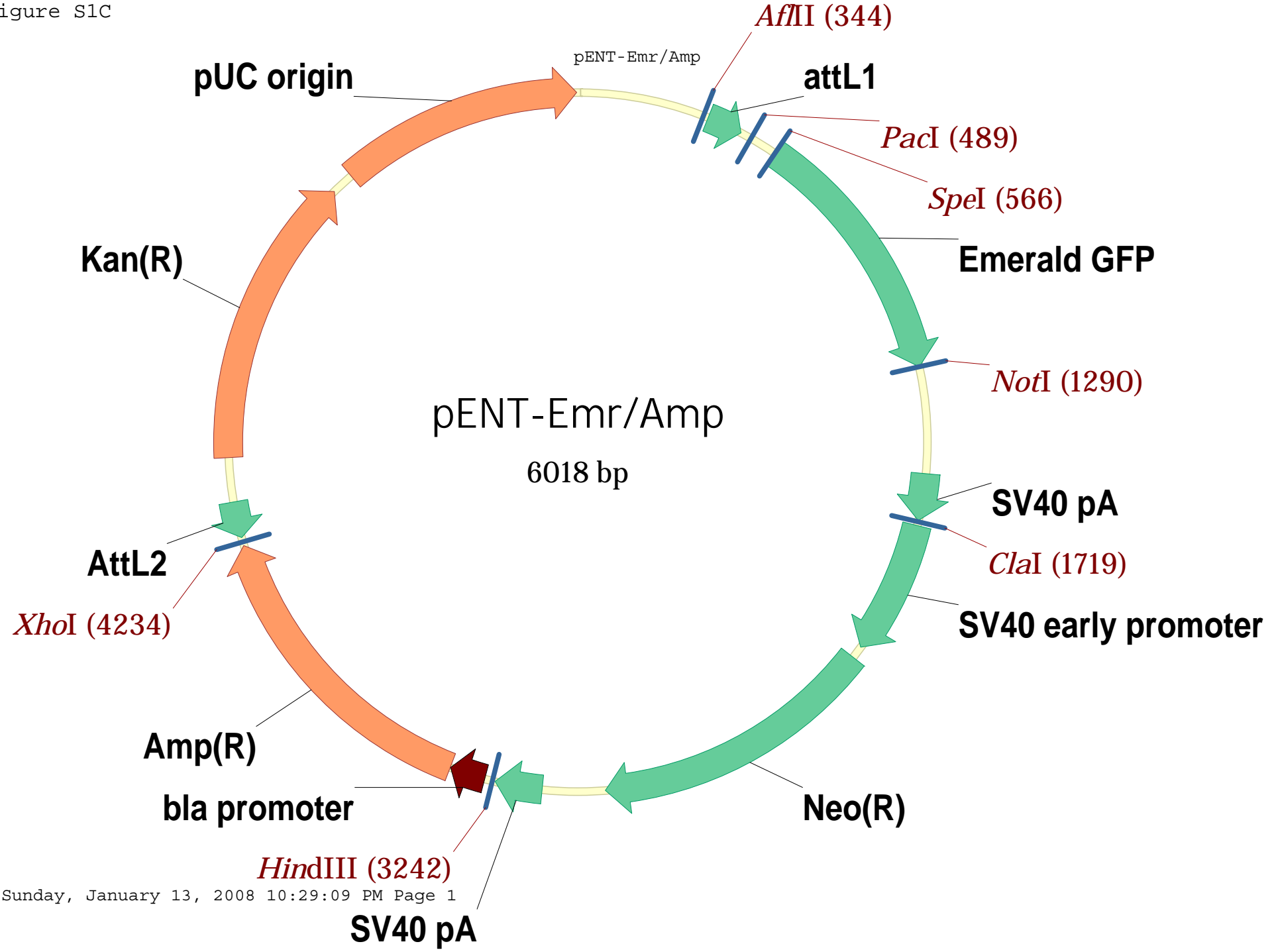

Figure S1D

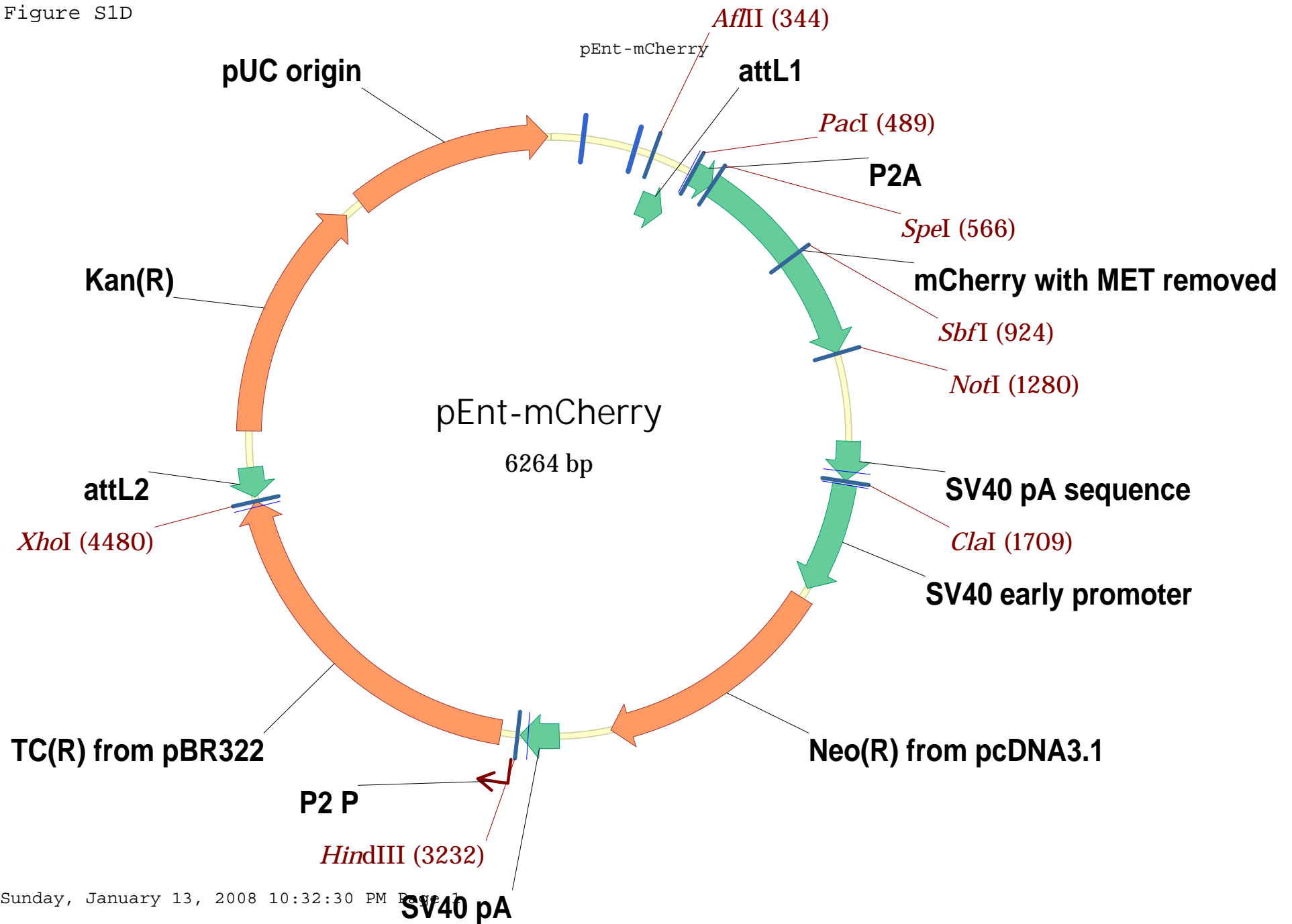

Figure S1E

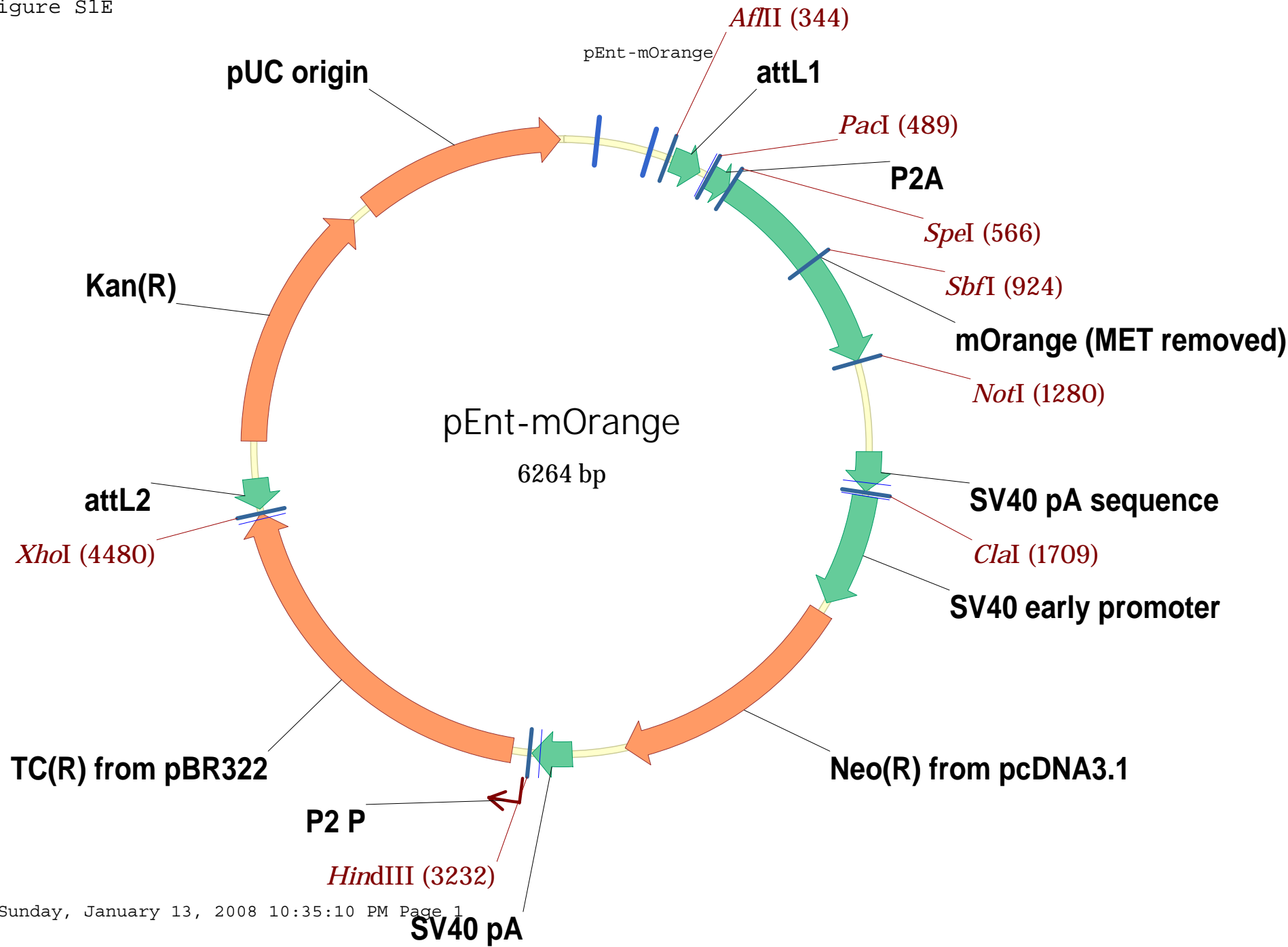

Figure S1F

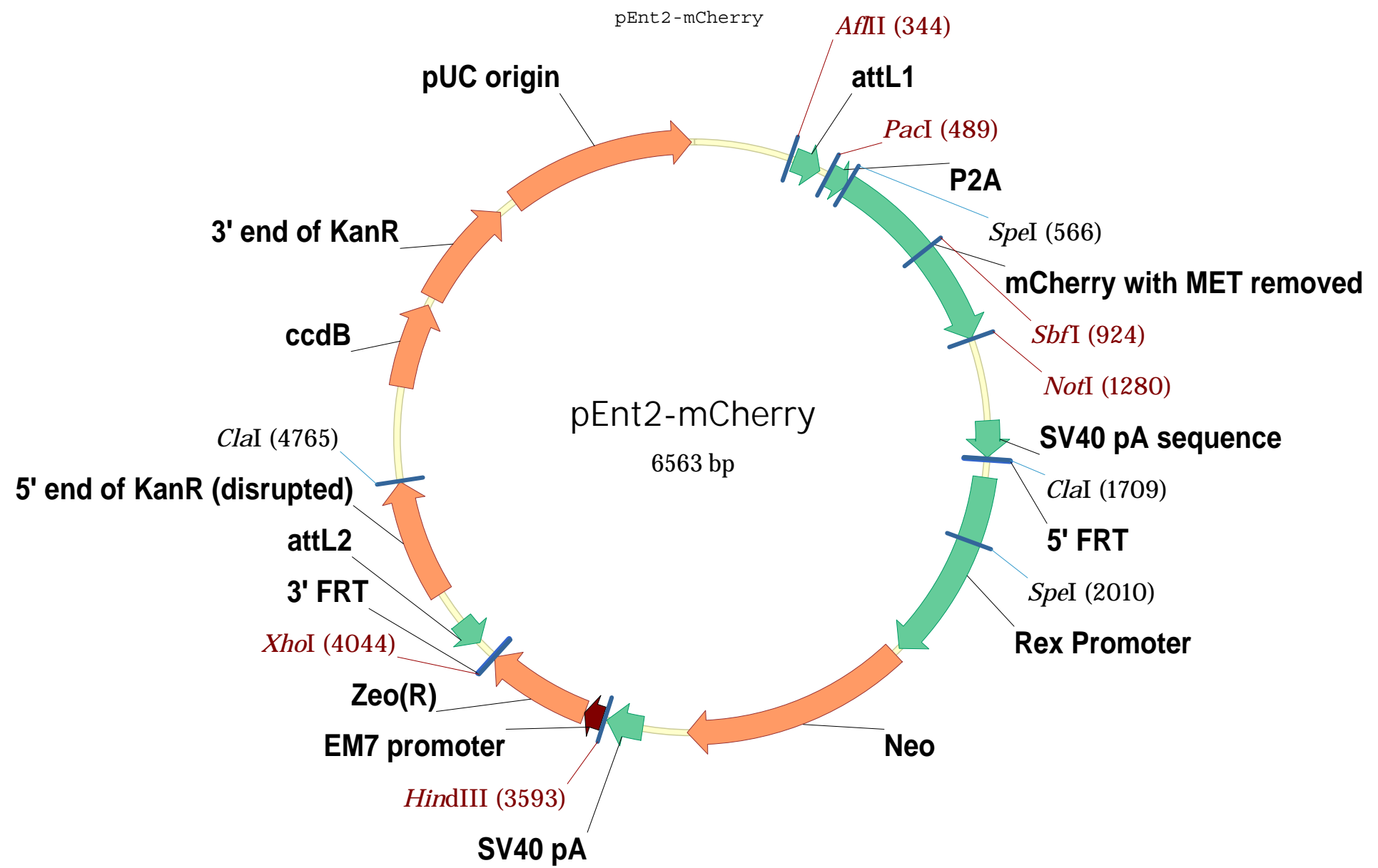

Supplement: Figure S1 — Maps of the different marker constructs with major features indicated. (A) pEnt-Emr/Tet and (B) pEnt-Emr/Zeo used in this manuscript. Unique restriction sites are noted in maroon, and restriction sites with more than one recognition sequence are in black. Additional marker cassettes have also been created, including (C) pEnt-Emr/Amp, (D) pEnt-mCherry, (E) pEnt-mOrange, and (F) pEnt2-mCherry. Note that pEnt2-mCherry contains a ccdB gene to increase the yield of correct targets after recombineering. In addition, a human Rex-1 promoter is used to drive the neomycin resistance gene. Both the eukaryotic and prokaryotic selection markers are flanked by FRT sites to allow optional excision. (0.07 MB PDF) [file pone.0002532.s001.pdf]

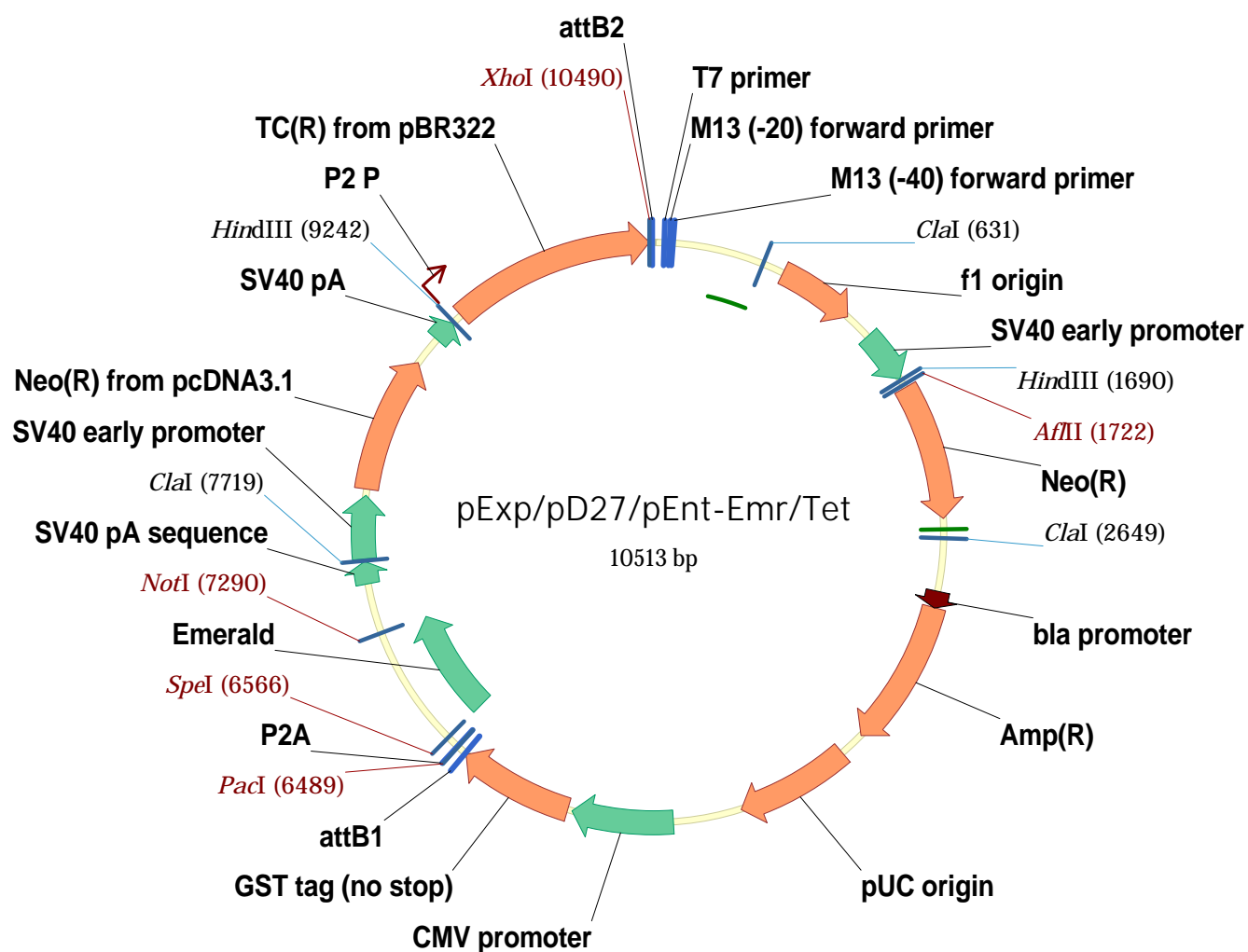

Supplement: Figure S2 — Map of pExp/pD27/pEnt-Emr expression vector indicating major features. Unique restriction sites are indicated in maroon, and restriction sites with more than one recognition sequence are in black. (0.02 MB PDF) [file pone.0002532.s002.pdf]

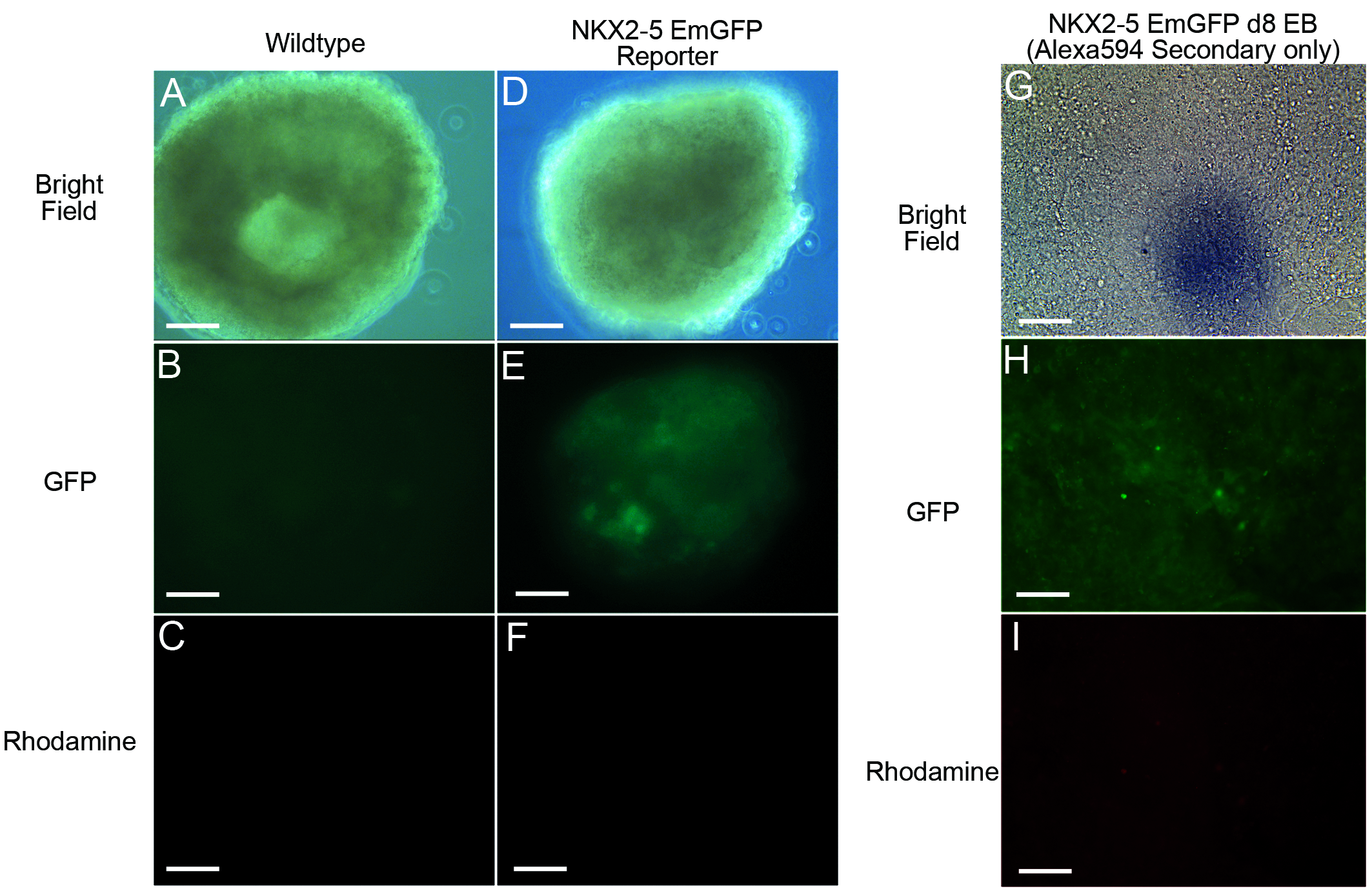

Supplement: Figure S4 — Immunohistochemistry background controls. Representative photographs of wild type E14 (A–C) and E14-NKX2-5-EmGFP (D–F) embryoid bodies examined at day 7 of differentiation show that NKX2-5 EmGFP fluorescence is easily detectable in the E14-NKX2-5-EmGFP EB, but no EmGFP fluorescence is present in the wild type EB. In addition, minimal auto-fluorescence in the rhodamine channel is detected in both the wild type and E14-NKX2-5-EmGFP lines. Twelve EBs derived from each line were examined. (G–I) Immunohistochemistry with the Alexa-594 secondary antibody, but no NKX2-5 primary antibody, on day 8 E14-NKX2-5-EmGFP embryoid bodies (after landing and attachment onto coverslips) show diffuse but detectable EmGFP (as previously seen in Fig. 2B) and no background staining by the Alexa-588 secondary antibody. White scale bars indicate 100 µm. (5.50 MB TIF) [file pone.0002532.s004.tif]
