# Supplementary material for: Marking Embryonic Stem Cells with a 2A Self-Cleaving Peptide: A NKX2-5 Emerald GFP BAC Reporter
Source: PLoS One. 2008 Jul 2;3(7):e2532. doi: 10.1371/journal.pone.0002532 (PMC2430532; doi:10.1371/journal.pone.0002532)
Supplement: Figure S3 — Maps of the modified region from the RP11-88L12 NKX2-5 BAC (RP11-88L12 NKX2-5-EmGFP). (A) Location of the human NKX2-5 open reading frame (blue, chr5: 172,591,744–172,594,868) within the RP11-88L12 BAC (black, chr5: 172,467,260–172,659,285 in NCBI Build 36.1). A second gene, BNIP1, was identified on RP11-88L12 by BAC-end sequencing (red, chr5: 172,504,146–172,523,950). (B) Enlargement of the modified NKX2-5 open reading frame showing insertion of the pEnt-Emr/Zeo marker cassette and locations of primers. (C) Sequence from the 5' junction showing locations of the 5' region of the NKX2-5 protein, 2A, primers, and emerald GFP. (0.03 MB PDF) [file pone.0002532.s003.pdf]

A

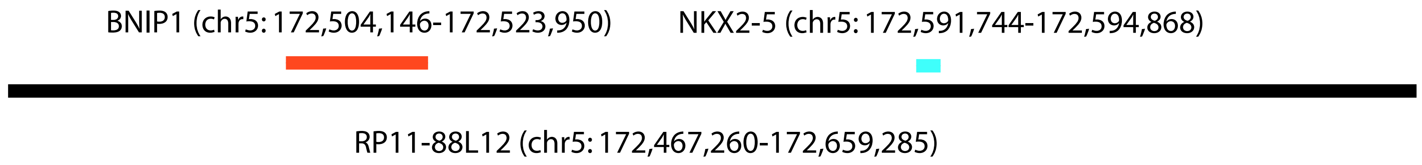

B

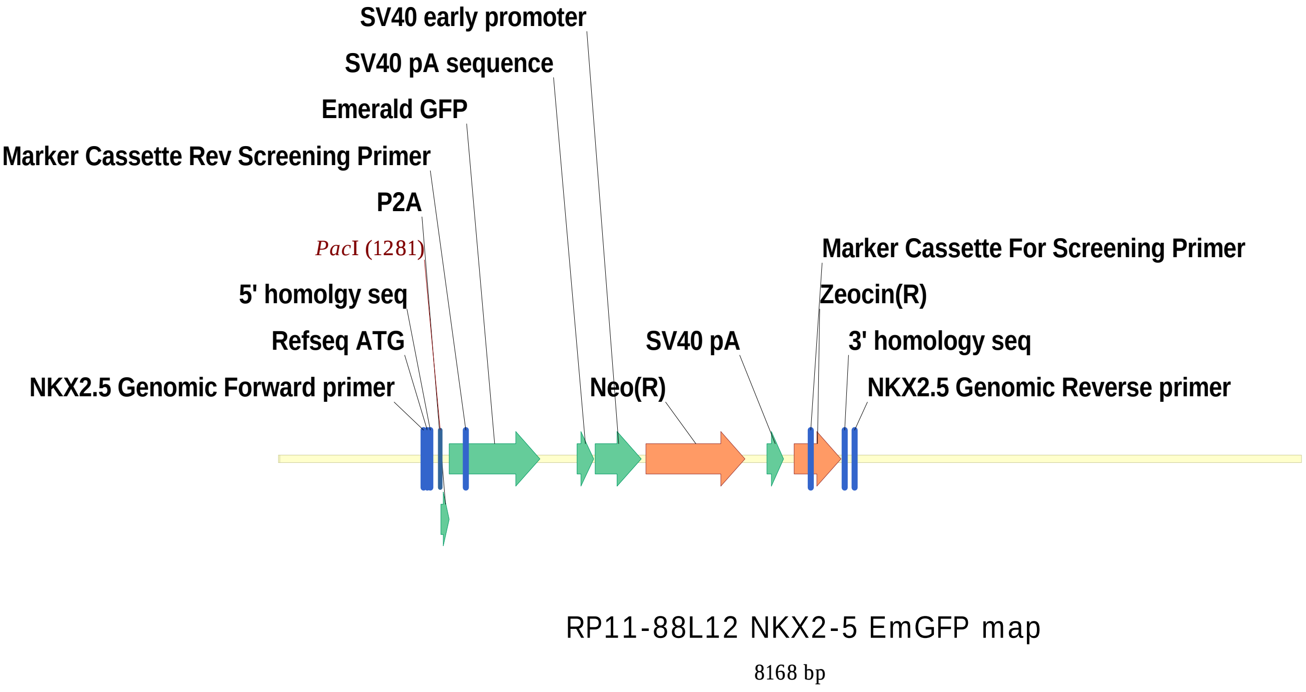

C

```
1001  GCCTGGTCCC GCCTCTCCTG CCCCTTGTGC TCAGCGCTAC CTGCTGCCCG GACACATCCA GAGCTGGCCG ACGGGTGCGC GGGCGGGCGG CGGCACCATG
      CGGACCAGGG CGGAGAGGAC GGGGAACACG AGTCGCGATG GACGACGGGC CTGTGTAGGT CTCGACCGGC TGCCCACGCG CCCGCCCGCC GCCGTGGTAC

                                                     NKX2.5 Genomic Forward primer              Refseq ATG
  +1                                                                                            Met  Phe  Pro Ser  Pro  Ala Leu  Thr
1101  CAGGGAAGCT GCCAGGGGCC GTGGGCAGCG CCGCTTTCTG CCGCCCACCT GGCGCTGTGA GACTGGCGCT GCCACCATGT TCCCCAGCCC TGCTCTCACG
      GTCCCTTCGA CGGTCCCCGG CACCCGTCGC GGCGAAAGAC GGCGGGTGGA CCGCGACACT CTGACCGCGA CGGTGGTACA AGGGGTCGGG ACGAGAGTGC

                              5' homolgy seq                                                PacI          P2A
  +1  Pro Thr Pro Phe Ser Val Lys Asp Ile Leu Asn Leu Glu Gln Gln Gln Arg Ser Leu Asn Gln Phe Ser Arg Gln Leu Ile Asn Val Glu Gly Ser Gly Ala·
1201  CCCACGCCCT TCTCAGTCAA AGACATCCTA AACCTGGAAC AGCAGCAGCG CAGCCTGAAC CAATTCAGTC GACAATTAAT TAACGTCGAG GGCAGCGGCG
      GGGTGCGGGA AGAGTCAGTT TCTGTAGGAT TTGGACCTTG TCGTCGTCGC GTCGGACTTG GTTAAGTCAG CTGTTAATTA ATTGCAGCTC CCGTCGCCGC

                                                                             Emerald GFP
                       P2A
  +1  ·Ala Thr Asn Phe Ser Leu Leu Lys Gln Ala Gly Asp Val Glu Glu Asn Pro Gly Pro Thr Ser Met Val Ser Lys Gly Glu Glu Leu Phe Thr Gly Val Val·
1301  CGACCAACTT TAGCCTGCTG AAACAGGCGG GCGATGTGGA AGAAAACCCA GGACCGACTA GTATGGTGAG CAAGGGCGAG GAGCTGTTCA CCGGGGTGGT
      GCTGGTTGAA ATCGGACGAC TTTGTCCGCC CGCTACACCT TCTTTTGGGT CCTGGCTGAT CATACCACTC GTTCCCGCTC CTCGACAAGT GGCCCCACCA

                                                     Emerald GFP
  +1  ·Val Pro Ile Leu Val Glu Leu Asp Gly Asp Val Asn Gly His Lys Phe Ser Val Ser Gly Glu Gly Glu Gly Asp Ala Thr Tyr Gly Lys Leu Thr Leu Lys
1401  GCCCATCCTG GTCGAGCTGG ACGGCGACGT AAACGGCCAC AAGTTCAGCG TGTCCGGCGA GGGCGAGGGC GATGCCACCT ACGGCAAGCT GACCCTGAAG
      CGGGTAGGAC CAGCTCGACC TGCCGCTGCA TTTGCCGGTG TTCAAGTCGC ACAGGCCGCT CCCGCTCCCG CTACGGTGGA TGCCGTTCGA CTGGGACTTC
                                                                                    Marker Cassette Rev Screening Primer

                                                     Emerald GFP
  +1  Phe Ile Cys Thr Thr Gly Lys Leu Pro Val Pro Trp Pro Thr Leu Val Thr Thr Leu Thr Tyr Gly Val Gln Cys Phe Ala Arg Tyr Pro Asp His Met Lys·
1501  TTCATCTGCA CCACCGGCAA GCTGCCCGTG CCCTGGCCCA CCCTCGTGAC CACCTTGACC TACGGCGTGC AGTGCTTCGC CCGCTACCCC GACCACATGA
      AAGTAGACGT GGTGGCCGTT CGACGGGCAC GGGACCGGGT GGGAGCACTG GTGGAACTGG ATGCCGCACG TCACGAAGCG GGCGATGGGG CTGGTGTACT
      Marker Cassette Rev Screening Primer
```
